# Supplementary material for: Quantitative lipidomic analysis of Ascaris suum
Source: PLoS Negl Trop Dis. 2020 Dec 2;14(12):e0008848. doi: 10.1371/journal.pntd.0008848 (PMC7710092; doi:10.1371/journal.pntd.0008848)
Supplement: Attachment — Submitted filename: Rejoinder_TW3_rbg1_rbg2.docx [file pntd.0008848.s008.docx]

***Editors of PLOS Neglected Tropical Diseases***

***Professors Sara Lustigman and Michael Cappello***

26 September 2020

**Dear Professors Lustigman and Cappello,**

**RE: Ms. Ref. No.: PNTD-D-20-01435_R1 entitled “Quantitative lipidomic analysis of *Ascaris suum*”**

**We thank you both for handling the manuscript, and the reviewers for their very constructive reports on our manuscript. In the following are our responses in a point-by-point manner (in bold-type):**

**Methods**

*-Are the objectives of the study clearly articulated with a clear testable hypothesis stated?
-Is the study design appropriate to address the stated objectives?
-Is the population clearly described and appropriate for the hypothesis being tested?
-Is the sample size sufficient to ensure adequate power to address the hypothesis being tested?
-Were correct statistical analysis used to support conclusions?
-Are there concerns about ethical or regulatory requirements being met?*

**Reviewer #1**: *Clearly described and appropriate.*
**RESPONSE: Thank you.**

**Reviewer #2**: *The methods are clear, logical and scientifically sound. However, some more description of how identifications were made would be helpful.*
**RESPONSE: Thank you. More description of how identifications were made has been added to the revised (R1) manuscript (page 9-10, lines 236-237 and 254-257).**

--------------------

**Results**
*-Does the analysis presented match the analysis plan?
-Are the results clearly and completely presented?
-Are the figures (Tables, Images) of sufficient quality for clarity?*

**Reviewer #1**: *Descriptive but comprehensive*
**RESPONSE: Thank you.**

**Reviewer #2**: *The results are interpreted in a logical fashion.*
**RESPONSE: Thank you.**

--------------------

**Conclusions**
*-Are the conclusions supported by the data presented?
-Are the limitations of analysis clearly described?
-Do the authors discuss how these data can be helpful to advance our understanding of the topic under study?
-Is public health relevance addressed?*

**Reviewer #1**: *The lipidomic analysis of Ascaris suum by Wang et al provides a unique qualitative characterization of various stages and tissue compartments of larval and adult male and female worms that is important as a baseline resource. The work is largely descriptive with no hypothesis testing but is nevertheless important in comparison to other free living and parasite nematode and helminth worms.
Specific criticisms:
The L4 and adult stages in the intestinal lumen have an acquired microbiome and, as luminal feeders, ingest host epithelial cells that are shed in large numbers into the intestine. The analysis does not attempt to distinguish host and microbiome lipids from that of the worm; which could be an important distinction in strategies to interfere with Ascaris development and residence in the host intestine.*

**RESPONSE: We thank the reviewer for raising this important issue. In mass spectrometry-based lipidomics, the identification of lipids is based on the mass-to-charge ratio of ions (generated from mass spectrometry), which reflects the chemical mass and structure of the lipids. Many lipid species (particularly lipid categories of GL, GP, ST and SM) with similar or the same mass-to-charge ratio of ions in mass spectrometry are found in different organisms, including bacteria, parasites and mammals. At this stage, there is no methodological approach that would allow the unequivocal distinction of host and/or microbiomic lipids from those of the worm. In contrast to the genomics and proteomics fields, there are limited public databases and resources available for comparative or ‘subtractive’ analyses/investigations. As lipidomics is an expanding field, it will take some time for well-defined data sets for a wide range of prokaryotic and eukaryotic taxa to be produced and for public databases to emerge which will allow unambiguous identification/delineation.**

**Although it might be challenging to eliminate the entire host-derived lipids from samples that harvested/collected from parasitic stages/sexes (e.g. gut content), we took extensive steps during the sample preparation (e.g., opening of alimentary tracts, and extensively washing; Methods section; page 6, lines 134-136) to eliminate or substantially reduce possible lipid ‘contaminants’. Thus, we are confident that residual host lipid contamination, if any, should not affect the main finding of substantial differences in the composition and abundance of lipids among developmental stages/sexes and organ systems of *Ascaris*. Moreover, it is possible that (active) intestinal uptake/absorption by the parasite itself would make differentiation of lipids of worm, host (chyme) and/or microbiomic origins extremely challenging once they have entered the worm. We have modified the *Methods* section of the revised manuscript R1 to describe these aspects to the reader (page 6, lines 151-156). In the present study, we made every effort to prepare representative samples to yield data that would underpin future lipidomic investigations.**

*The pseudo-coelomic or peri-enteric fluid of adult worms is a major source of lipid containing molecules that may differ between adult male and female worms, but it was not characterized in this study. This is curious since it is readily available and easy to isolate. Please provide an explanation for the absence of distinct data on this worm compartment.*
**RESPONSE: We thank the reviewer for raising this issue. The reason for not including the suggested experiment is that the *semi-quantitative* comparison of the current analytical approach is to normalise the detected MS ions intensity to the dry weight of samples. In order to compare quantitative data among stages and organ systems of *Ascaris*, it would not be possible to collect enough dry weight (4 mg) of these fluids for lipid extraction. If qualitative analyses had been carried out, this could have been done, but not for a quantitative or semi-quantitative comparative analysis. Nonetheless, we are eager to undertake a separate study to characterise the body and intestinal fluids of adult worms in the future.**

**Reviewer #2**: *The conclusions are justified by the data presented.*
**RESPONSE: Thank you.**
--------------------

**Editorial and Data Presentation Modifications?**
Use this section for editorial suggestions as well as relatively minor modifications of existing data that would enhance clarity. If the only modifications needed are minor and/or editorial, you may wish to recommend “Minor Revision” or “Accept”.

**Reviewer #1**: *minor revisions*
**RESPONSE: Thank you.**

**Reviewer #2**: *I only have a few suggestions for the authors to consider.
1. It might be a good idea to define the authors meaning of "identified" when the term is first used.*

**RESPONSE: Thank you for the suggestion. More explanation on how lipids were “identified” has been provided in the revised manuscript (page 9-10, lines 236-237 and 254-257).**

*2. In the Methods or Results section some description of how identifications were confirmed would be helpful for the reader to be able to assess the reliability of "confident identifications". I am left guessing as to how identifications were made other than by measured mass. What type of fragments were matched in MS/MS spectra and to what?*

**RESPONSE: Apologies for the confusion. LipidSearch was used to search a ‘large’ database of > 1.5 million lipid ions and their predicted fragment ions. Identification of lipids (classes) and acyl/alkyl chains were established based on m/z values of lipid precursor and acyl/alkyl chain and class-specific fragmentation product ions using LipidSearch (page 9-10, lines 236-237 and 254-257).**

**The reason for using “confidently” identified and quantified in the *Methods* and *Results* sections is that we wanted to emphasise that we used a stringent cut-off (i.e. every LC-MS feature was manually inspected and re-integrated, and at least 2 replicates were required). We have modified the text to clarify these points.**

*3. I am ignorant of the lipidome of nematodes, but do they make sterols? Perhaps some comment regarding other lipid classes which were not analysed is warranted, particularly as there is much discussion of membrane fluidity.*
**RESPONSE: We thank the reviewer for raising this issue. Nematodes do make sterols, and we have included cholesterol ester (CE) from sterols category as an available class for identification via LipidSearch. However, *Ascaris* CEs are present in very minute amounts and were not recorded due to the stringent cut-off criteria that needed to be set in this first study (pages 9 and 10, lines 237-265). The lipid classes recorded are presented in Table S1 in the revised manuscript.**

*Minor points.
1. What was the logic behind adding BHT at the very end of the sample preparation protocol?*

**RESPONSE: We thank the reviewer for raising this issue. BHT is an antioxidant and was added to prevent lipid autooxidation during extraction. We have modified the text to address this issue (page 7, lines 168-177).**

*2. For those not using Thermo instruments it would be good learn the difference between CID and HCD.*

**RESPONSE: Thank you for the suggestion. More information of CID and HCD has been added to the *Methods* section (page 8-9, lines 217-218 and 230-231).**  *3. Similarly, some explanation of top-3 data dependent MS3 scans would be useful.*
**RESPONSE: We thank the reviewer for raising this issue. More information of MS^3^ scans has been added to the *Methods* section (page 9, lines 230-231).**

--------------------

**Summary and General Comments**
Use this section to provide overall comments, discuss strengths/weaknesses of the study, novelty, significance, general execution and scholarship. You may also include additional comments for the author, including concerns about dual publication, research ethics, or publication ethics. If requesting major revision, please articulate the new experiments that are needed.

**Reviewer #1**: (No Response)

**Reviewer #2**: A nice paper but it could be improved by provision of more details regarding lipid identification.

**RESPONSE: Thank you. The information on lipid identification has been addressed (page 9-10, lines 236-237 and 254-257).**

**CONCLUSION**

**We hope that we have satisfactorily addressed all of the issues raised by the reviewers and that the manuscript PNTD-D-20-01435_R1 now meets the requirements for publication in *PLOS Neglected Tropical Diseases*. We look forward to your decision.**

On behalf of all authors,

Robin B. Gasser,

Robin B. Gasser | Redmond Barry Distinguished Professor | The University of Melbourne, Australia | E: [robinbg@unimelb.edu.au](https://protect-au.mimecast.com/s/amnICvl0E5uZ0VJNHQiKvy?domain=applewebdata) |

M: +61 0430 6060 48 |
